# Metal–Oleate Complex-Derived Bimetallic Oxides Nanoparticles Encapsulated in 3D Graphene Networks as Anodes for Efficient Lithium Storage with Pseudocapacitance

**DOI:** 10.1007/s40820-019-0247-3

**Published:** 2019-02-25

**Authors:** Yingying Cao, Kaiming Geng, Hongbo Geng, Huixiang Ang, Jie Pei, Yayuan Liu, Xueqin Cao, Junwei Zheng, Hongwei Gu

**Affiliations:** 1 Key Laboratory of Organic Synthesis of Jiangsu Province, College of Chemistry, Chemical Engineering and Materials Science and Collaborative Innovation Center of Suzhou Nano Science and Technology, Soochow University, Suzhou, 215123 People’s Republic of China; 2 School of Chemical Engineering and Light Industry, Guangdong University of Technology, Guangzhou, 510006 People’s Republic of China; 3 School of Chemical and Biomedical Engineering, Nanyang Technological University, Singapore, 637459 Singapore; 4 College of Physics, Optoelectronic and Energy, Soochow University, Suzhou, 215006 People’s Republic of China

**Keywords:** Metal–oleate complex, Bimetallic oxides nanoparticles, Porous architecture, 3D graphene networks, Lithium ion batteries

## Abstract

In this manuscript, we have demonstrated the delicate design and synthesis of bimetallic oxides nanoparticles derived from metal–oleate complex embedded in 3D graphene networks (MnO/CoMn_2_O_4_ ⊂ GN), as an anode material for lithium ion batteries. The novel synthesis of the MnO/CoMn_2_O_4_ ⊂ GN consists of thermal decomposition of metal–oleate complex containing cobalt and manganese metals and oleate ligand, forming bimetallic oxides nanoparticles, followed by a self-assembly route with reduced graphene oxides. The MnO/CoMn_2_O_4_ ⊂ GN composite, with a unique architecture of bimetallic oxides nanoparticles encapsulated in 3D graphene networks, rationally integrates several benefits including shortening the diffusion path of Li^+^ ions, improving electrical conductivity and mitigating volume variation during cycling. Studies show that the electrochemical reaction processes of MnO/CoMn_2_O_4_ ⊂ GN electrodes are dominated by the pseudocapacitive behavior, leading to fast Li^+^ charge/discharge reactions. As a result, the MnO/CoMn_2_O_4_ ⊂ GN manifests high initial specific capacity, stable cycling performance, and excellent rate capability.

**Electronic supplementary material:**

The online version of this article (10.1007/s40820-019-0247-3) contains supplementary material, which is available to authorized users.

## Highlights

- Bimetallic oxides nanoparticles derived from metal–oleate complexes embedded in 3D graphene networks were fabricated by a facile and rational design approach.
- The unique porous architecture promotes charge transfer so as to enhance the reversible capacity.
- The synergetic effect between the 0D nanoparticles and 3D graphene networks plays an essential role in the superb electrochemical performance.

**Electronic supplementary material:**

The online version of this article (10.1007/s40820-019-0247-3) contains supplementary material, which is available to authorized users.

## Graphical abstract

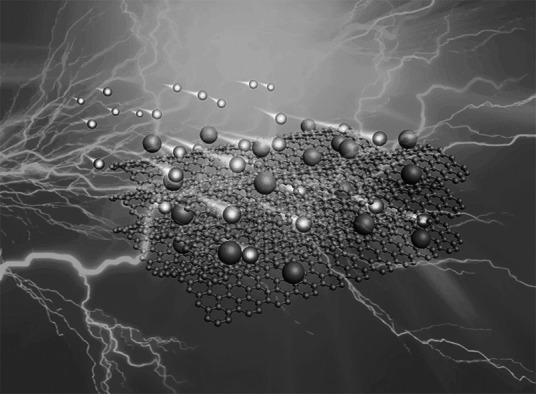

## Introduction

In recent years, there has been enormous interest for lithium ion batteries (LIBs) due to their significant merits such as small volume, long cycle life, high specific capacity, and good safety [1–3], which are applicable in various fields such as digital products, satellites, and portable mobile tools [4–7]. Compared to the traditional anode material (graphite has a low specific capacity of 372 mAh g^−1^) [8–10], transition metal oxides (TMOs) can deliver a higher theoretical specific capacity (500–1000 mAh g^−1^) that is promising to replace the conventional graphite material in LIBs [11, 12].

Among the examined TMO materials, cobalt oxide has attracted considerable attention for its high specific capacity of 890 mAh g^−1^ [13–15]. However, the structure variation caused by volume expansion during Li^+^ insertion and extraction processes hinders its practical application [16–18]. As is well known, the addition of manganese into cobalt oxide structure to form cobalt–manganese binary oxides could enrich the overall valence electrons of the hybrid material, leading to enhanced electrical conductivity of the anode and thus, improve the electrochemical performance of the LIBs [19]. In addition to that, manganese in its oxide form not only possesses an extremely low operating voltage [20–22], but is also a plentiful and low-cost material which is beneficial for practical implementation [23, 24]. Interestingly, bimetallic oxides, such as CoMn_2_O_4_ [25], MnCo_2_O_4_ [26], ZnCo_2_O_4_ [27], ZnFe_2_O_4_ [28], exhibit excellent electrochemical performance owing to their complementarity and synergetic effect during the charge and discharge processes. In addition, graphene is one of the most promising templates used for supporting nanoparticles for application in LIBs [29, 30]. The dispersion of metal oxides nanoparticles on graphene-based materials could enhance the electrical conductivity and buffer volume variation of the electrode and could further improve the electrochemical performance of LIBs [31]. For instance, Yang et al. [32] presented a facile strategy for the synthesis of graphene-coated Co_3_O_4_ fiber electrode, which exhibits excellent cyclic stability and good rate capacity. Wang et al. [33] prepared graphene-coated Mn_3_O_4_ with unprecedented rate capability and cycling stability, which was attributed to the intimate interaction between graphene substrates and Mn_3_O_4_ nanoparticles.

Herein, we present a facile and rational design approach to fabricate MnO/CoMn_2_O_4_ ⊂ GN composite, in which the MnO/CoMn_2_O_4_ nanoparticles are uniformly wrapped by graphene sheets, which are connected into a three-dimensional (3D) conductive network. The electrochemical performance of the as-prepared MnO/CoMn_2_O_4_ ⊂ GN electrode was evaluated in LIBs, which exhibits excellent electrochemical performance, such as stable cycling performance (about 921 mAh g^−1^ over 150 cycles at 0.1 A g^−1^) and good rate capability (about 515 mAh g^−1^ at 5 A g^−1^).

## Experimental

### Synthesis of the CoMn_2_O_4_ Nanoparticles

In a typical procedure, CoCl_2_·6H_2_O (3 mM, 0.7138 g), MnCl_2_·4H_2_O (9 mM, 1.7811 g), and sodium oleate (24 mM, 7.3068 g) were dissolved in a mixture which contained 18 mL of water, 24 mL of ethanol and 42 mL of hexane, and then stirred at 70 °C for 4 h. The organic layer was separated with a separatory funnel and washed three times with water. Afterward, the as-obtained precursor was dried at 80 °C for 12 h under vacuum conditions. To synthesize CoMn_2_O_4_ nanoparticles, the precursor was calcined at 350 °C under Ar atmosphere for 1 h and then heated up to 600 °C in air for 3 h.

### Synthesis of the MnO/CoMn_2_O_4_ ⊂ GN Nanocomposite

The as-prepared CoMn_2_O_4_ nanoparticles (80 mg) were dispersed in 25 mL of deionized water via ultrasonication for 30 min. A modified Hummer’s method was used to synthesize graphene oxide (GO) from natural graphite powder. Then, the as-obtained GO suspension was further sonicated for several hours to exfoliate the graphene oxide layers which were then dispersed in water at a concentration of 2 mg mL^−1^ [34]. Next, GO aqueous solution (40 mg, 2 mg mL^−1^) was added into the above suspension and stirred for 6 h. The mixture was transferred and sealed in a Teflon-lined stainless steel autoclave, while keeping the reaction temperature at 180 °C for 12 h. After cooling down to the room temperature, the resulting product was washed with ethanol and water three times and then sintered at 500 °C under Ar atmosphere for 2 h. Comparable samples were prepared by a similar procedure with different feed ratios displayed in Table S1. As we can see, the sample with the higher content of Mn/GO is denoted as M1/G1, and the sample with the lower content of Mn/GO is denoted as M2/G2.

### Materials Characterization

The morphology of the product was examined by scanning electron microscopy (SEM, Hitachi S-4700), transmission electron microscopy (TEM, Tecnai G220, FEI), and high-resolution TEM (HRTEM, Tecnai G2 F20 S-TWIN). The elemental constituents were characterized by energy-dispersive X-ray spectroscopy (EDS, Hitachi S-4700). The crystallographic information was analyzed by X-ray diffraction (XRD) on a X’Pert-Pro MPD diffractometer (PANalytical, Netherlands) with a Cu Kα X-ray source (*λ* = 1.540598 Å). Thermogravimetric analysis (TGA) was performed on PerkinElmer TGA 4000 thermogravimetric analyzer, and X-ray photoelectron spectroscopy (XPS, Escalab250Xi, UK) was conducted with a hemispherical electron energy analyzer. The specific surface area was performed via a Brunauer–Emmett–Teller (BET, Micromeritics ASAP 2020 M) analyzer, and the pore size distribution was calculated through the Barrett–Joyner–Halenda (BJH) method.

### Electrochemical Measurement

The electrochemical performances of the products were measured using a coin-type half cell (CR 2016). Active materials (MnO/CoMn_2_O_4_ ⊂ GN, 70  wt%), acetylene black (20  wt%), and polyvinylidene difluoride (PVDF, 10  wt%) were dissolved in N-methyl-2-pyrrolidone (NMP) to form a uniform slurry. Then, the slurry was spread on the copper foil as the working electrode and dried in a vacuum oven at 85 °C overnight. The mass loading was around 1.4 mg cm^−2^ on each current collector. The electrode sheets were then pressed under a force of approximately 10 MPa and cut into circular sheets. A cell was assembled in an argon-filled glove box with a lithium foil as the reference electrode, copper foil with dried anode materials as the working electrode, while a Celgard 2400 membrane served as the separator immersed in the electrolyte containing 1 M LiPF_6_ in a mixed solution of ethylene carbonate (EC) and diethyl carbonate (DEC) with a volume ratio of 1:1. A LAND CT2001 test system was employed to perform the electrochemical measurements. The cyclic voltammetry (CV) test was carried out at a sweep rate of 0.1 mV s^−1^ with a voltage window of 0.01–3.0 V.

## Results and Discussion

**Scheme 1:**
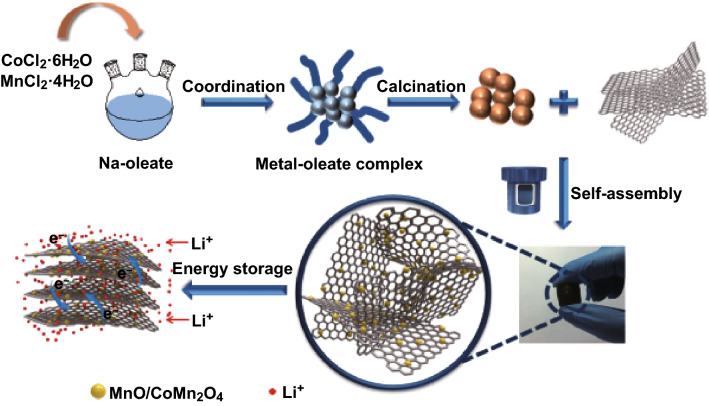
Schematic illustration of the synthetic processes for MnO/CoMn_2_O_4_ ⊂ GN

The multistep fabrication procedure of the MnO/CoMn_2_O_4_ ⊂ GN is schematically illustrated in Scheme 1. The CoMn_2_O_4_ nanoparticles are obtained after thermal decomposition of the metal–oleate precursors, which were prepared by reacting CoCl_2_·6H_2_O, MnCl_2_·4H_2_O and sodium oleate in mixed solvents at 70 °C for 4 h, stirring overnight and spin drying. Subsequently, the as-prepared CoMn_2_O_4_ nanoparticles were assembled with graphene sheets by a hydrothermal process and post-heat treatment, yielding MnO/CoMn_2_O_4_ ⊂ GN composite.

**Fig. 1:**
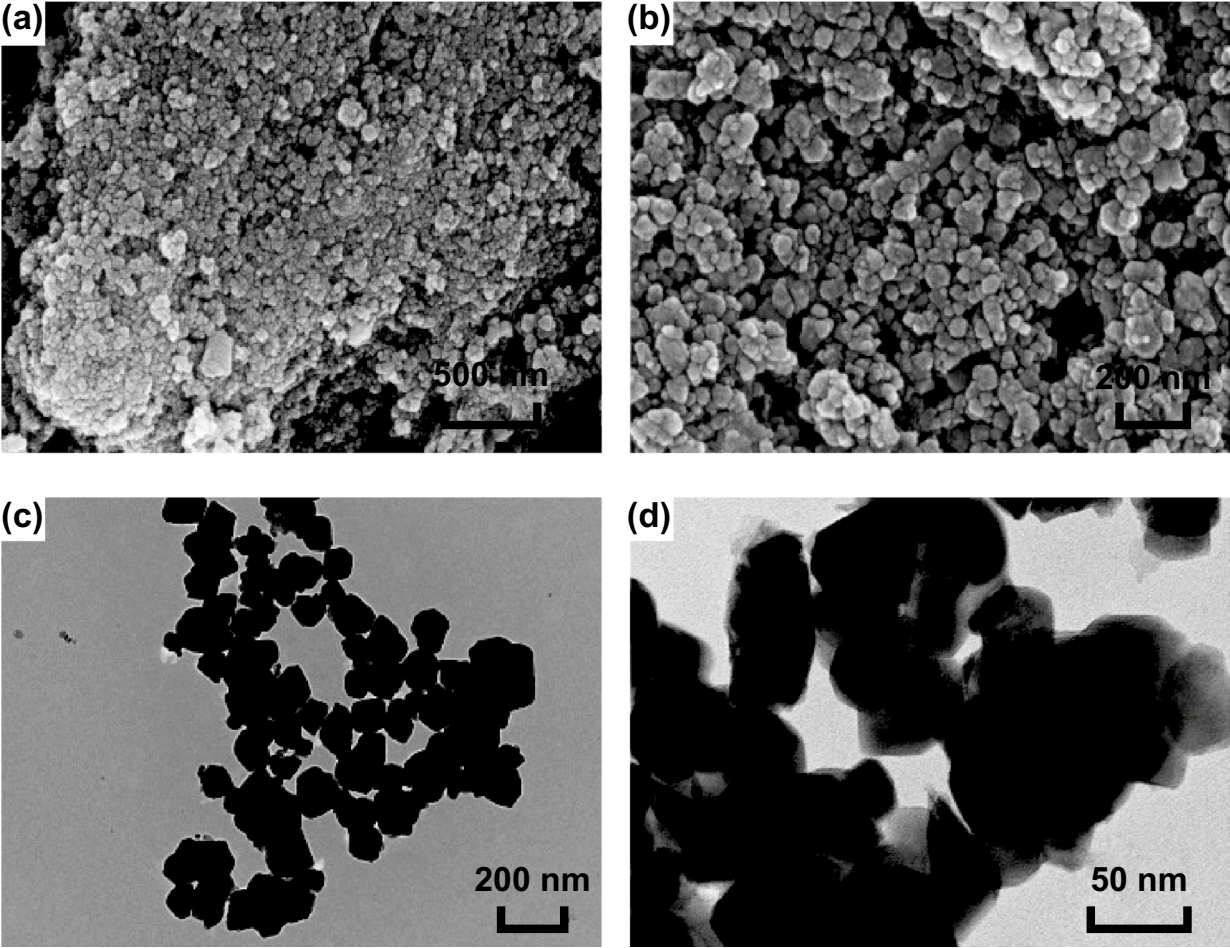
**a**, **b** SEM and **c**, **d** TEM images of the CoMn_2_O_4_

The morphologies and structures of the CoMn_2_O_4_ sample were investigated by SEM and TEM. The SEM (Fig. 1a, b) and TEM (Fig. 1c, d) images reveal that the cobalt–manganese bimetallic oxides are composed of nanoparticles with diameters ranging from 50 to 100 nm. The optimal calcination temperature to form CoMn_2_O_4_ was determined based on the TGA (Fig. S1), performed at a temperature range from 25 to 800 °C with a heating rate of 10 °C min^−1^ under N_2_ and air atmosphere. The weight loss between 10 and 350 °C under N_2_ (red) might be ascribed to the removal of adsorbed water. The decomposition temperature of metal–oleate is about 500 °C under air atmosphere (black). The existence and contents of the elements in the as-synthesized CoMn_2_O_4_ nanoparticles can be characterized by EDS (Fig. S3a and Table S2).

**Fig. 2:**
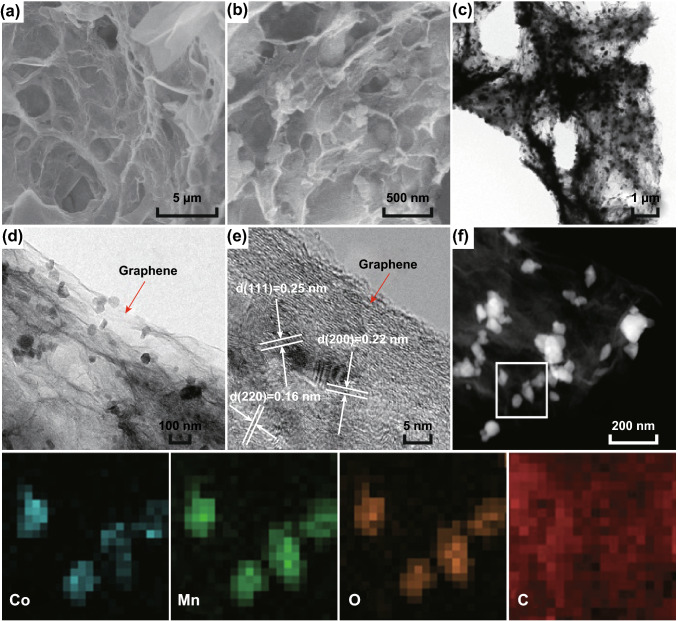
**a**, **b** SEM and **c**, **d** TEM images of MnO/CoMn_2_O_4_ ⊂ GN. **e** High-magnification TEM image of MnO/CoMn_2_O_4_ ⊂ GN. **f** The corresponding elemental mapping images of the MnO/CoMn_2_O_4_ ⊂ GN for Co, Mn, O, and C

The MnO/CoMn_2_O_4_ ⊂ GN was obtained by embedding CoMn_2_O_4_ in 3D graphene networks through a self-assembly method. As shown in Fig. 2a, b, the MnO/CoMn_2_O_4_ nanoparticles are uniformly wrapped by rough and irregular graphene networks. Further, TEM investigations reveal that almost all 0D nanoparticles are coated with graphene sheets which are connected into 3D graphene networks (Fig. 2c, d). The existence of 3D graphene networks is also confirmed by Raman spectra in Fig. S2. The G band is a characteristic feature of graphitic layers, while the D band corresponds to disordered carbon or defective graphitic structures. The intensity ratio of the D and G peak is widely used as a metric of disorder in graphene [35]. Thus, the high *I*_D_/*I*_G_ in MnO/CoMn_2_O_4_ ⊂ GN (1.13) demonstrates a more disordered structure compared to reduced graphene oxide (rGO) (*I*_D_/*I*_G_ = 0.98), which is advantageous for enhancing the electrical conductivity [36]. Benefit from these structures is that the volume expansion can be suppressed during Li^+^ charge/discharge so as to improve the cycling performance of LIBs [37]. The HRTEM image shows a *d*-spacing value of 0.22 nm, which can be attributed to (200) plane of MnO. The other clear lattice spacings of about 0.25 and 0.16 nm correspond to the (111) and (220) planes of CoMn_2_O_4_, respectively. The elemental mapping images of MnO/CoMn_2_O_4_ ⊂ GN are displayed in Fig. 2f. It can be seen that the elements of Co, Mn, and O are highly homogeneously distributed, while C is primarily dispersed outside. This clearly shows that the MnO/CoMn_2_O_4_ nanoparticles are incorporated in the rGO layers. The above mapping results are in accordance with EDS spectra of MnO/CoMn_2_O_4_ ⊂ GN in Fig. S3b and Table S3.

**Fig. 3:**
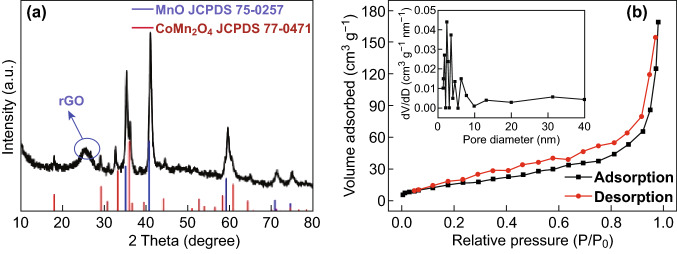
**a** XRD pattern of the MnO/CoMn_2_O_4_ ⊂ GN, **b** Nitrogen adsorption–desorption isotherms and corresponding pore size distribution plot of the MnO/CoMn_2_O_4_ ⊂ GN

The crystallographic structure and composition of the MnO/CoMn_2_O_4_ ⊂ GN samples are characterized by XRD (Fig. 3a). The weak peak at around 25^o^ assigned to the reduction in GO to rGO, which is marked in Fig. 3a. The spectrum undoubtedly reveals the formation of the CoMn_2_O_4_ with a tetragonal crystal structure (JCPDS No. 77-0471) and MnO with a cubic crystal structure (JCPDS No. 75-0257) [38]. In addition, the XRD analysis of pure CoMn_2_O_4_ was carried out for comparison and is shown in Fig. S4a. All the dominant diffraction peaks are well matched with the (202), (113), (311), and (404) planes of CoMn_2_O_4_ (JCPDS No. 18-0408) [39]. Further, the crystal structure of the sample changes after being coated with graphene sheets. The transformation of the crystallographic structure might be due to the fact that part of the bimetallic oxides is reduced during the hydrothermal process. The specific surface area and pore size of MnO/CoMn_2_O_4_ ⊂ GN are calculated by the Brunauer–Emmett–Teller (BET) method and Barrett–Joyner–Halenda (BJH) method, respectively, as shown in Fig. 3b. The MnO/CoMn_2_O_4_ ⊂ GN possesses a specific surface area of 136.6 m^2^ g^−1^ and an average pore size of 2.7 nm. As compared to MnO/CoMn_2_O_4_ ⊂ GN, the BET and BJH methods show that the CoMn_2_O_4_ nanoparticles exhibit a smaller specific surface area (31.7 m^2^ g^−1^) and a narrower pore size (2.1 nm), as shown in Fig. S4b.

**Fig. 4:**
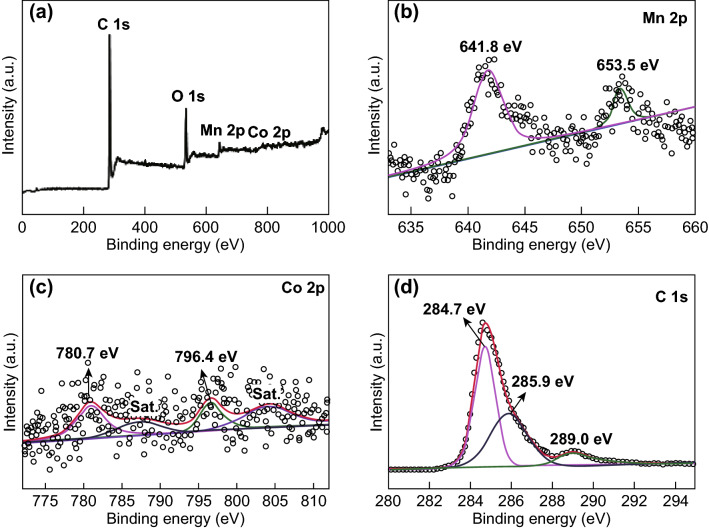
XPS spectra for the MnO/CoMn_2_O_4_ ⊂ GN composites: **a** the survey spectrum and the high-resolution spectra for **b** Mn 2p, **c** Co 2p and **d** C 1s

The chemical composition and electronic properties of the obtained MnO/CoMn_2_O_4_ ⊂ GN were further characterized by XPS. It can be found in Fig. 4 that the XPS survey spectrum confirms the existence of Mn, Co, and C elements in the sample. The Mn 2p XPS spectrum (Fig. 4b) contains two main spin–orbit peaks of Mn 2p_3/2_ at 641.8 eV and Mn 2p_1/2_ at 653.5 eV, respectively, corresponding to the presence of both Mn^2+^ and Mn^3+^ [40]. By using a Gaussian fitting method, the Co 2p spectrum (Fig. 4c) is best fitted to two spin–orbit peaks and two shake-up satellites (denoted as sat.). The two major peaks located at 780.7 and 796.4 eV correspond to the Co 2p_3/2_ and Co 2p_1/2_ states with a splitting spin–orbit separation of 15.7 eV, which confirms the presence of Co^2+^ [39]. The XPS spectrum of C 1s (Fig. 4d) consists of three peaks centered at 284.7, 285.9, and 289 eV which can be assigned to C–C, C–O, and O–C=O chemical bands, respectively [41]. According to Fig. S5, when coated with reduced graphene oxides, the contents of C in MnO/CoMn_2_O_4_ ⊂ GN increased significantly.

**Fig. 5:**
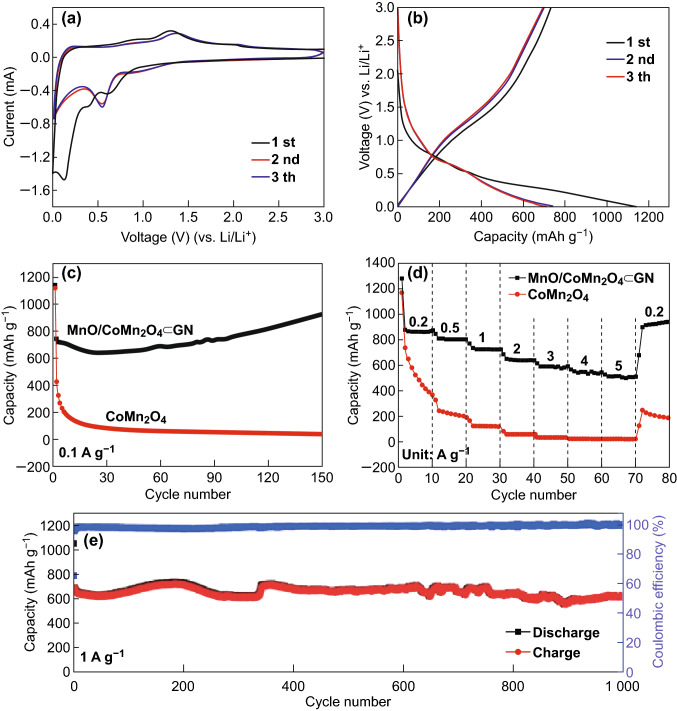
**a** CV profile of MnO/CoMn_2_O_4_ ⊂ GN at a scan rate of 0.1 mV s^−1^ in the voltage range of 0.01–3.0 V. **b** The charge and discharge profiles of MnO/CoMn_2_O_4_ ⊂ GN. **c** Cycling performance and Coulombic efficiency of the CoMn_2_O_4_ and MnO/CoMn_2_O_4_ ⊂ GN electrodes at a current density of 0.1 A g^−1^. **d** Rate performances of the CoMn_2_O_4_ and MnO/CoMn_2_O_4_ ⊂ GN electrodes at various current densities. **e** Cycling performance of the MnO/CoMn_2_O_4_ ⊂ GN electrode at a current density of 1 A g^−1^

To evaluate the electrochemical performances of the MnO/CoMn_2_O_4_ ⊂ GN anode material in LIBs, CV measurements of the MnO/CoMn_2_O_4_ ⊂ GN anode were conducted at a voltage range of 0.01–3.0 V versus Li/Li^+^ with a scan rate of 0.1 mV s^−1^ (Fig. 5a). It is obvious that the CV curve for the first cycle is different from those of the subsequent ones. In the first scan, the peak in the range of 0.1–0.4 V can be attributed to the reduction in MnO/CoMn_2_O_4_ to metallic Mn and Co, respectively [42]. The minor broad peak at around 0.75 V can be attributed to the formation of a solid electrolyte interphase (SEI) layer, which is generated by the irreversible decomposition of the solvent in the electrolyte [43]. In the anodic process, two broad peaks at 1.31 and 2.01 V correspond to the oxidation of metallic Mn and Co [44], respectively. In the subsequent cycles, two pairs of redox peaks at 0.54/1.38 V and 1.02/2.01 V correspond to the reduction/oxidation in the oxide of Mn and Co, respectively [37]. From the second cycle onward, the CV plots overlap well with each other, demonstrating good reversibility and excellent stability of the MnO/CoMn_2_O_4_ ⊂ GN materials [45]. The galvanostatic charge and discharge profiles of MnO/CoMn_2_O_4_ ⊂ GN electrode for the first, second, and third cycles at a current density of 0.1 A g^−1^ in the potential range from 0.01 to 3.0 V are shown in Fig. 5b. The MnO/CoMn_2_O_4_ ⊂ GN electrode possesses higher initial discharge and charge capacities of 1140 and 735 mAh g^−1^, respectively, compared to the corresponding theoretical values (921 mAh g^−1^) [45]. The extra capacity at the first discharge may be due to the formation of the SEI layers [46]. According to calculations, the initial Coulombic efficiency is about 64.5%. The Coulombic efficiency increases as the cycles growing up and is then continuously maintained around 99%.

The cycling performances of MnO/CoMn_2_O_4_ ⊂ GN and pure CoMn_2_O_4_ compared at a current density of 0.1 A g^−1^ are shown in Fig. 5c. The specific discharge capacity of MnO/CoMn_2_O_4_ ⊂ GN exhibits a slight decrease in the first 24 cycles, followed by a gradual increase to 921 mAh g^−1^ during the 150th cycle. This phenomenon occurs in many transition metal oxides, owing to the high rate lithiation-induced reactivation [47, 48]. The MnO/CoMn_2_O_4_ ⊂ GN exhibits a high reversible specific capacity of 921 mAh g^−1^ after 150 cycles, with Coulombic efficiency increased to about 99%. A similar phenomenon can be seen in Fig. 5e and S6 regarding the long-term cycling performance of the MnO/CoMn_2_O_4_ ⊂ GN electrode at high current densities of 1 and 5 A g^−1^. After 1000 cycles, the reversible capacity of MnO/CoMn_2_O_4_ ⊂ GN can still remain 622 mAh g^−1^ with its Coulombic efficiency around 100%, confirming the high architectural stability. As a comparison, the cycling performance of pure CoMn_2_O_4_ is shown in Fig. S7. Obviously, the CoMn_2_O_4_ sample exhibits poor electrochemical properties, delivering a lower initial discharge capacity (1125 mAh g^−1^) than MnO/CoMn_2_O_4_ ⊂ GN, which reduces drastically to 59 mAh g^−1^ after 200 cycles. When increasing or decreasing the content of Mn and GO in Table S1, the electrochemical performances will all be worse, as can be seen in Fig. S8. The outstanding cycling performance of MnO/CoMn_2_O_4_ ⊂ GN might be the result of the unique graphene-coating design. The 3D graphene networks can serve as a conductive matrix to enlarge the interface contact between the active material and electrolyte and shorten the diffusion pathway for lithium ions effectively [41]. In addition, as a compliant buffer, the flexible graphene sheets can accommodate the volume change and prevent the aggregation of MnO/CoMn_2_O_4_ nanoparticles during the charge and discharge process [31, 49]. Due to the above reasons, enhanced cycle stability and high reversible capacity can be obtained. In order to further comprehend the electrochemical performance, the rate performances of the CoMn_2_O_4_ and MnO/CoMn_2_O_4_ ⊂ GN electrodes are also shown in Fig. 5d. It can be seen that the discharge capacities of MnO/CoMn_2_O_4_ ⊂ GN are 870.6, 809.6, 731.0, 647.5, 590.4, 545.7, and 515.3 mAh g^−1^ at current densities of 0.2, 0.5, 1, 2, 3, 4, and 5 A g^−1^, respectively. Moreover, the discharge capacity can deliver up to 914.5 mAh g^−1^ when the current density is back to 0.2 A g^−1^, revealing remarkable cycling stability and rate capability. In contrast, at current densities of 0.2, 0.5, 1, 2, 3, 4, and 5 A g^−1^, the capacities of the pure CoMn_2_O_4_ are 649.0, 235.7, 127.8, 61.1, 32.5, 21.1, and 20.8 mAh g^−1^, respectively. Thus, the rate capacity of MnO/CoMn_2_O_4_ ⊂ GN is much superior to that of the CoMn_2_O_4_. The morphology and structure of the MnO/CoMn_2_O_4_ ⊂ GN electrode material after 150 cycles at a current density of 0.1 A g^−1^ are displayed in Fig. S9. In view of the well-preserved surface features, it can be confirmed that MnO/CoMn_2_O_4_ ⊂ GN has a stable structure due to the protective effect of the graphene conductive networks.

**Fig. 6:**
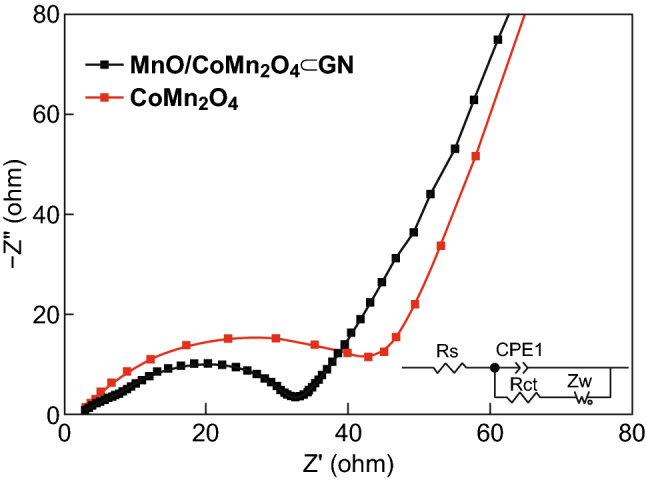
Electrochemical impedance spectroscopy plots of pure CoMn_2_O_4_ and MnO/CoMn_2_O_4_ ⊂ GN electrodes

Electrochemical impedance spectroscopy (EIS) of the CoMn_2_O_4_ and MnO/CoMn_2_O_4_ ⊂ GN was further investigated as shown in Fig. 6. The MnO/CoMn_2_O_4_ ⊂ GN has a smaller diameter of the depressed semicircle at high frequencies compared to the pure CoMn_2_O_4_ in the EIS curves. This result clearly explains that the charge transfer resistance of MnO/CoMn_2_O_4_ ⊂ GN is smaller during the charge and discharge processes, leading to improved electrode reaction kinetics and better cycling performance.

**Fig. 7:**
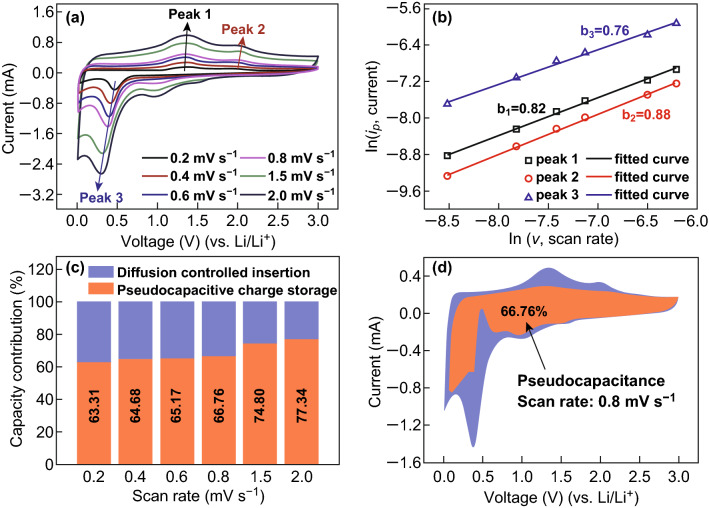
**a** CV profiles of the MnO/CoMn_2_O_4_ ⊂ GN electrodes at different scan rates with the potential range between 0.01 and 3.0 V. **b** The fitted lines and ln(*i*_*p*_*)* versus ln(*v*) plots at different oxidation and reduction states. **c** The percentages of pseudocapacitive contribution at different scan rates. **d** Pseudocapacitive (red) and diffusion-controlled (blue) contribution to the charge storage of MnO/CoMn_2_O_4_ ⊂ GN at 0.8 mV s^−1^. (Color figure online)

To further understand the nature of the charge storage process, kinetic study based on CV analysis is carried out. Figure 7a shows the CV measurements of MnO/CoMn_2_O_4_ ⊂ GN at different scanning speeds of 0.2, 0.4, 0.6, 0.8, 1.5, and 2 mV s ^−1^. The curves show analogous shapes with three redox peaks during cathodic and anodic processes at each scan rate. It is well known that the capacitance and diffusion-controlled contributions to the whole capacity can be determined by Eqs. 1 and 2 [50]:

1 $$i = av^{b}$$

2 $$\ln i = b\ln v + \ln a$$

where *i* is the peak current, *v* is potential sweep rate, *a* and *b* are adjustable parameters. The *b* value is determined by the slope of the ln(*v*) − ln(*i*) plots according to Eq. 2. When the *b* value is close to 0.5 or 1.0, the electrochemical system is controlled by ion-diffusion or pseudocapacitance, respectively. From Fig. 7b, the *b* values for the anodic and cathodic peaks are 0.82, 0.88, and 0.76, implying that the pseudocapacitive behavior primarily controls the electrochemical processes of MnO/CoMn_2_O_4_ ⊂ GN electrode, resulting in high rate capabilities [51]. It can be seen that the calculated *b* values of MnO/CoMn_2_O_4_ ⊂ GN are higher than those of pure CoMn_2_O_4_ and rGO electrodes in Figs. S10 and S11, suggesting that the pseudocapacitance effect is more significant in MnO/CoMn_2_O_4_ ⊂ GN compared to pure CoMn_2_O_4_ and rGO. We can use another analysis to further quantify the capacitive contribution to the total lithium storage [52]. The current response *i* at a certain potential *V* could be separated into pseudocapacitive effects (*k*_*1*_*v)* and diffusion-controlled reactions (*k*_*2*_*v*^*0.5*^), according to Eqs. 3 and 4:

3 $$i = k_{1} v + k_{2} v^{0.5}$$

4 $$i/v^{0.5} = k_{1} v^{0.5} + k_{2}$$

where the *k*_*1*_ and *k*_*2*_ are fixed for the same electrochemical reaction. Figure 7c illustrates the calculated capacitive contribution at various scan rates. It can be found that the percentage of capacitive contribution keeps increasing with increase in the scan rate from 0.2 to 2 mV s^−1^, which well explains the superior rate capability. The typical voltage profile for the dominating capacitive contributions (red region) in comparison with the whole area is shown in Fig. 7d. At a scan rate of 0.8 mV s^−1^, the pseudocapacitive contribution is calculated to be 66.76%, suggesting that the capacity contribution is dominated by pseudocapacitance. It can be observed that the capacitive capacity keeps increasing along with the increase in voltage scan rates, and pseudocapacitive contribution is more dominant in the total capacity for MnO/CoMn_2_O_4_ ⊂ GN compared to pure CoMn_2_O_4_ and rGO.

**Fig. 8:**
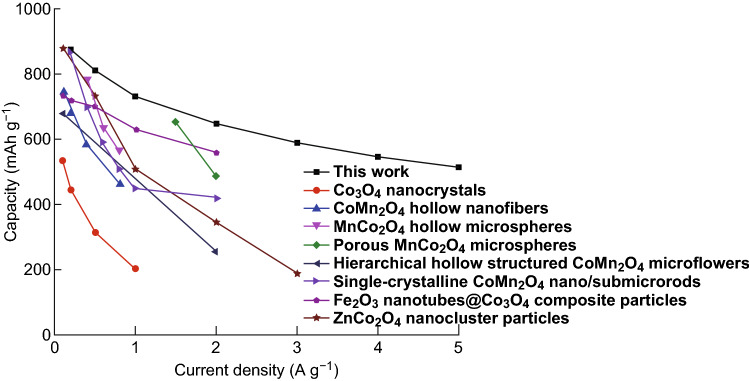
Comparison of the lithium storage properties of the MnO/CoMn_2_O_4_ ⊂ GN to similar anode materials reported in other studies

To highlight the electrochemical performances of the MnO/CoMn_2_O_4_ ⊂ GN in this work, other similar electrode materials reported in the previous literature are also compared and presented in Fig. 8. According to the line chart, the as-prepared MnO/CoMn_2_O_4_ ⊂ GN electrode can deliver a higher current density when comparing with other similar anode materials reported in recent studies like Co_3_O_4_ nanocrystals [48], CoMn_2_O_4_ hollow nanofibers [39], MnCo_2_O_4_ hollow microspheres [45], porous MnCo_2_O_4_ microspheres [47], hierarchical hollow structured CoMn_2_O_4_ microflowers [53], single-crystalline CoMn_2_O_4_ nano/submicrorods [54], Fe_2_O_3_ nanotubes@Co_3_O_4_ composite particles [55], and ZnCo_2_O_4_ nanocluster particles [56], indicating that the unique architecture of bimetallic oxides nanoparticles encapsulated in 3D graphene networks can largely enhance the electrochemical performance of anode materials.

As mentioned above, the pure CoMn_2_O_4_ nanoparticles can be synthesized via metal–oleate decomposition method rapidly and on a large scale. The synthetic procedure is a general approach to synthesize different kinds of transition metal oxides. The porous MnO/CoMn_2_O_4_ ⊂ GN is fabricated by coating the MnO/CoMn_2_O_4_ with rGO through a self-assembly process. As an anode material for LIBs, the MnO/CoMn_2_O_4_ ⊂ GN displays superior and excellent electrochemical performances which could be attributed to the following factors. Firstly, the unique porous architecture promotes the charge transfer so as to enhance the reversible capacity by shortening the diffusion length of the lithium ion [57]. Secondly, the presence of 3D graphene networks can not only facilitate the transport of lithium ions and electrons but also buffer the large volume change of anodes and alleviate the pulverization problem, leading to excellent cyclic stability. Furthermore, the synergetic effect between the 0D nanoparticles and 3D graphene networks plays an essential role in the superb electrochemical performances of MnO/CoMn_2_O_4_ ⊂ GN electrode. The void space in the MnO/CoMn_2_O_4_ ⊂ GN can facilitate electron and ion transportation. The 3D graphene networks can also improve the electrical conductivity and help to increase the mechanical strength, leading to superior electrochemical performance, such as high specific capacity, outstanding cycle stability, and excellent rate capability.

## Conclusions

In summary, we synthesize a novel architecture consisting of 0D nanoparticles encapsulated in 3D interconnected graphene networks by a self-assembly route. When used as an anode for LIBs, the unique MnO/CoMn_2_O_4_ ⊂ GN nanocomposites hold several structural and compositional advantages including improved electrical conductivity, reduced diffusion length for Li^+^ ions, minimized volume variation, and increased number of active sites for electrochemical reactions. The MnO/CoMn_2_O_4_ ⊂ GN anode exhibits a pseudocapacitance-boosted ultrafast lithium storage performance in terms of high capacity and good cycling stability (921 mAh g^−1^ over 150 cycles at 0.1 A g^−1^), as well as outstanding rate capability (about 515 mAh g^−1^ at 5 A g^−1^). This study may inspire the design and construction of advanced anode materials for LIBs.

## Electronic supplementary material

Below is the link to the electronic supplementary material.

Supplementary material 1 (PDF 756 kb)
