# Supplementary material for: Metal–Oleate Complex-Derived Bimetallic Oxides Nanoparticles Encapsulated in 3D Graphene Networks as Anodes for Efficient Lithium Storage with Pseudocapacitance
Source: Nanomicro Lett. 2019 Feb 25;11:15. doi: 10.1007/s40820-019-0247-3 (PMC7770734; doi:10.1007/s40820-019-0247-3)
Supplement: Supplementary file 1 — Supplementary material 1 (PDF 756 kb) [file 40820_2019_247_MOESM1_ESM.pdf]

Supporting Information for

# Metal-Oleate Complex Derived Bimetallic Oxides Nanoparticles Encapsulated in 3D Graphene Networks as Anodes for Efficient Lithium Storage with Pseudocapacitance


Yingying Cao[1, #], Kaiming Geng[1, #], Hongbo Geng[2, *], Huixiang Ang[3], Jie Pei[1], Yayuan Liu[1], Xueqin Cao[1], Junwei Zheng[4], Hongwei Gu[1, *]

[1]Key Laboratory of Organic Synthesis of Jiangsu Province, College of Chemistry, Chemical Engineering and Materials Science and Collaborative Innovation Center of Suzhou Nano Science and Technology, Soochow University, Suzhou 215123, People's Republic of China

[2]School of Chemical Engineering and Light Industry, Guangdong University of Technology, Guangzhou 510006, People's Republic of China

[3]School of Chemical and Biomedical Engineering, Nanyang Technological University, Singapore 637459, Singapore

[4]College of Physics, Optoelectronic and Energy, Soochow University, Suzhou 215006, People's Republic of China

[#]These authors contributed equally to this work

*Corresponding authors. E-mail: hongwei@suda.edu.cn (Hongwei Gu); hbgeng@gdut.edu.cn (Hongbo Geng)


## Supplementary Figures and Tables

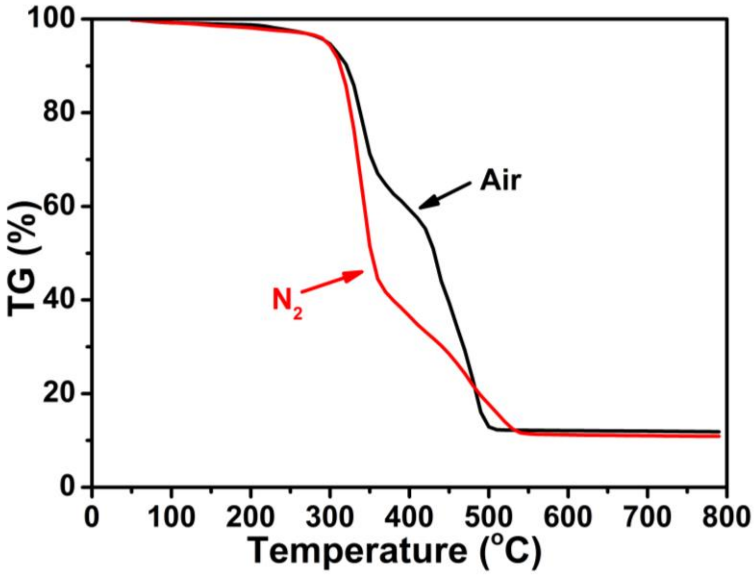


**Fig. S1** TGA plot of the metal-oleate complex precursor under $N_2$ and air atmospheres

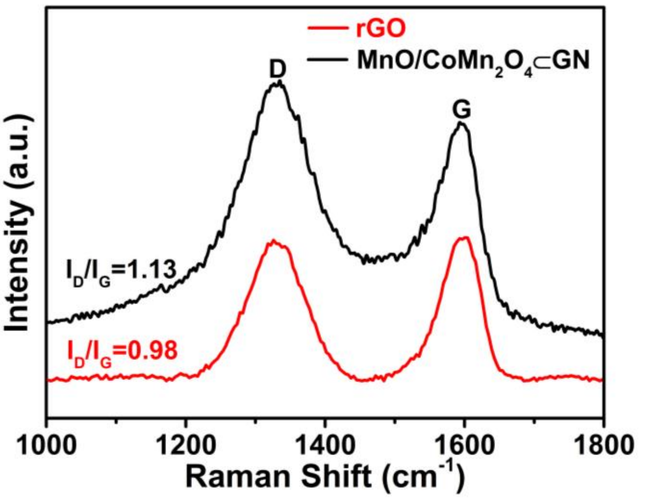


**Fig. S2** Raman spectra of MnO/CoMn$_2$O$_4$⊂GN and rGO

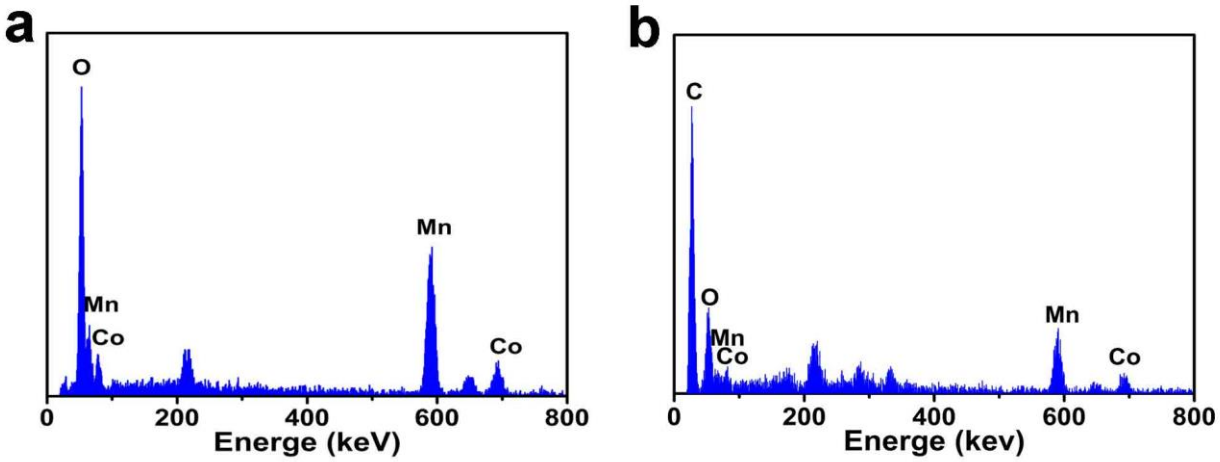


**Fig. S3** Energy-dispersive X-ray spectroscopy (EDS) plots of CoMn$_2$O$_4$ and MnO/CoMn$_2$O$_4$⊂GN

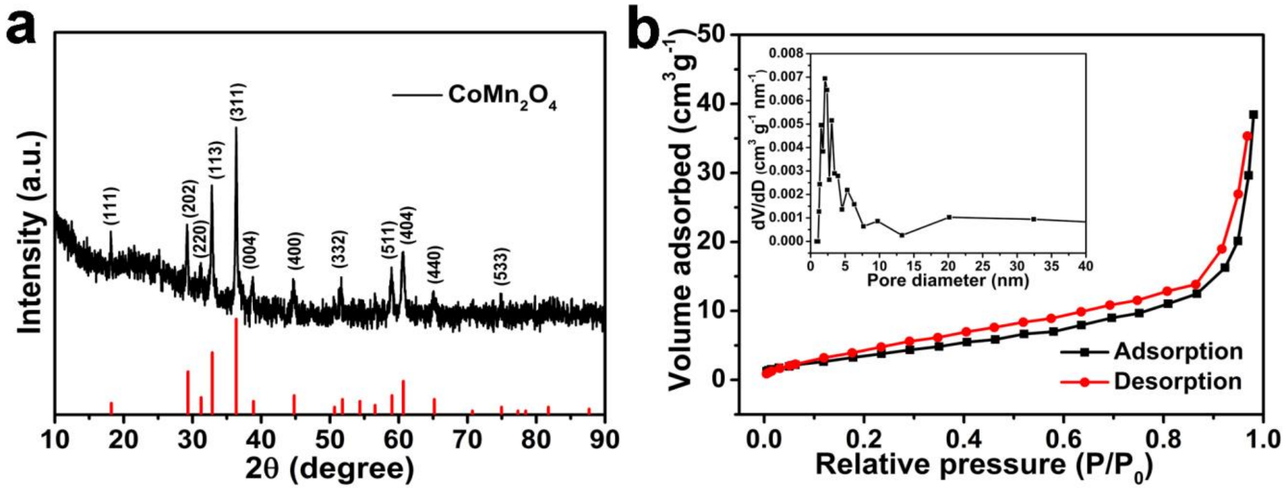


**Fig. S4 a** XRD pattern of the CoMn$_2$O$_4$. **b** Nitrogen adsorption-desorption isotherms and the pore size distribution curve of the CoMn$_2$O$_4$

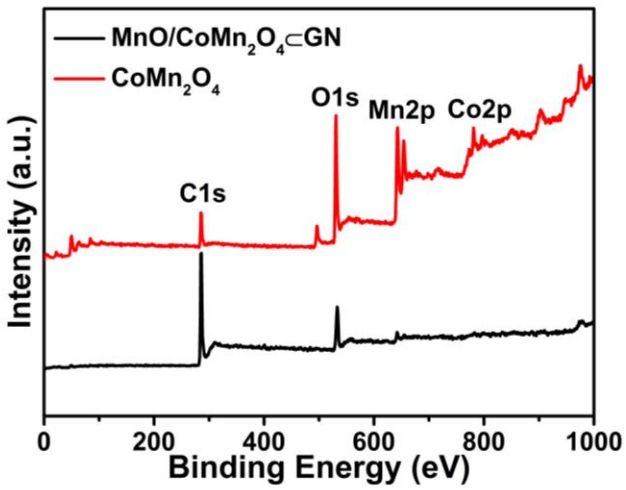


**Fig. S5** XPS spectra of the $CoMn_2O_4$ and $MnO/CoMn_2O_4$⊂GN samples

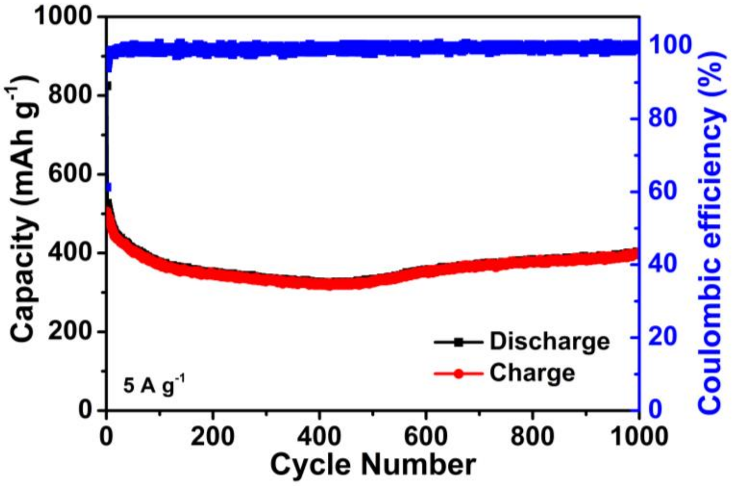


**Fig. S6** Cycling performance and coulombic efficiency of $MnO/CoMn_2O_4$⊂GN at a current density of 5 A $g^{-1}$

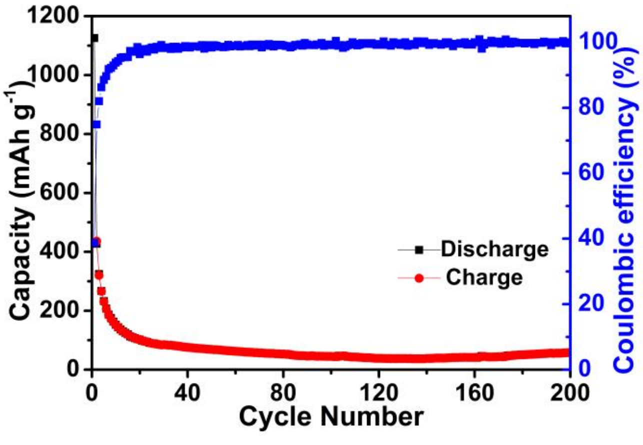


**Fig. S7** Cycling performance and coulombic efficiency of pure $CoMn_2O_4$ at a current density of 0.1 A $g^{-1}$

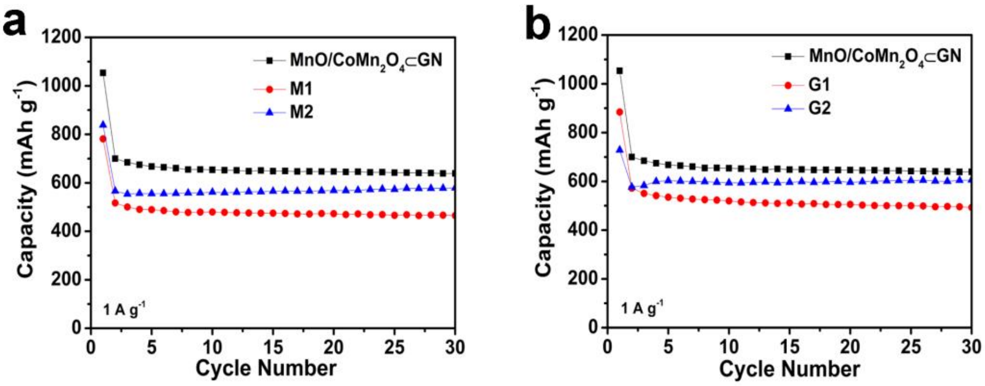


**Fig. S8** Cycling performances of MnO/$CoMn_2O_4$⊂GN, M1, M2, G1, and G2 at a current density of 1 A $g^{-1}$

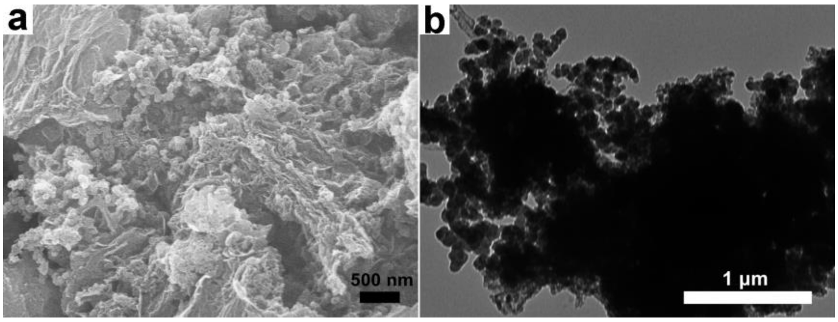


**Fig. S9 a** SEM and **b** TEM images of MnO/$CoMn_2O_4$⊂GN after 150 cycles at 0.1 A $g^{-1}$

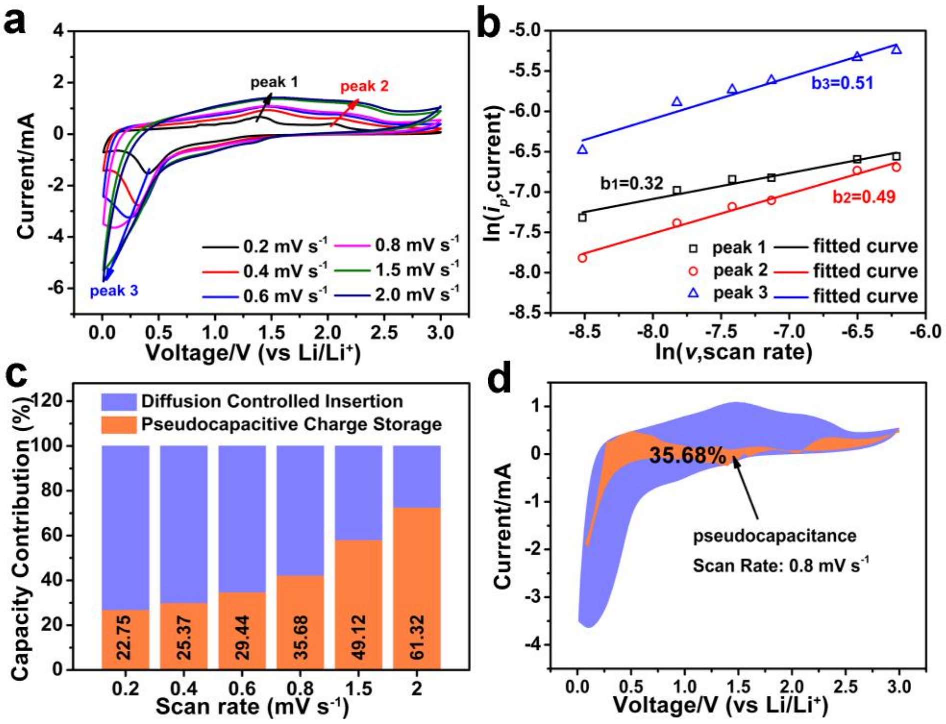


**Fig. S10 a** CV profiles of the pure $CoMn_2O_4$ electrodes at different scan rates with the potential range between 0.01 and 3.0 V. **b** The fitted lines and ln($i_p$) *versus* ln($v$) plots at different oxidation and reduction states. **c** The percentages of pseudocapacitive contribution at different scan rates. **d** Pseudocapacitive (red) and diffusion-controlled (blue) contribution to the charge storage of $CoMn_2O_4$ at 0.8 mV $s^{-1}$

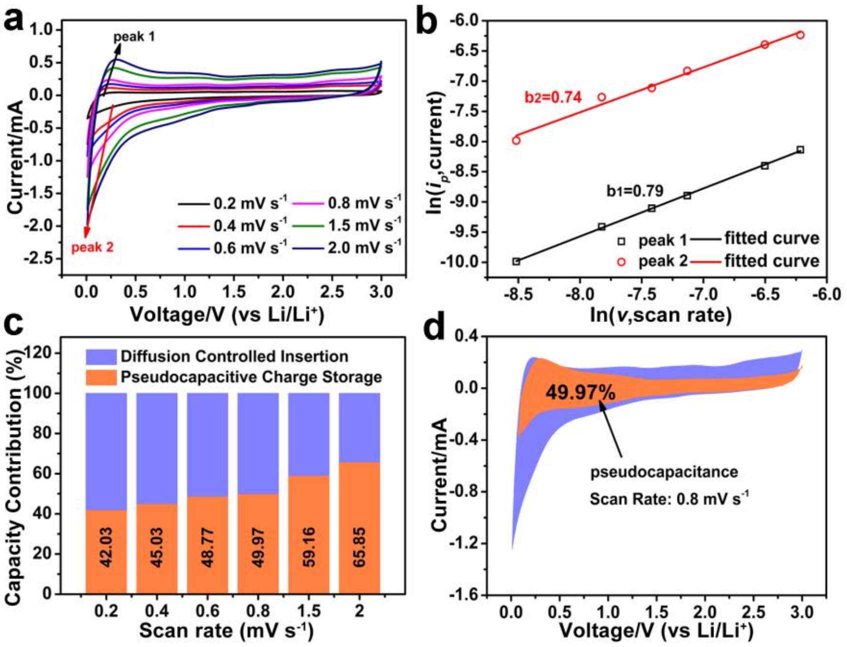


**Fig. S11 a** CV profiles of the rGO electrodes at different scan rates with the potential range between 0.01 and 3.0 V. **b** The fitted lines and ln($i_p$) *versus* ln($v$) plots at different oxidation and reduction states. **c** The percentages of pseudocapacitive contribution at different scan rates. **d** Pseudocapacitive (red) and diffusion-controlled (blue) contribution to the charge storage of rGO at 0.8 mV $s^{-1}$

**Table S1** The feed ratio of M1, M2, G1, and G2

| | $CoCl_2{\cdot}6H_2O$ (mmol) | $MnCl_2{\cdot}4H_2O$ (mmol) | GO (mg) |
|---|---|---|---|
| $MnO/CoMn_2O_4{\subset}GN$ | 3.0 | 9.0 | 40 |
| M1 | 1.5 | 9.0 | 40 |
| M2 | 3.0 | 4.5 | 40 |
| G1 | 3.0 | 9.0 | 80 |
| G2 | 3.0 | 9.0 | 20 |

**Table S2** The content of each component of $CoMn_2O_4$

| Element | Co K | Mn K | O K | Total |
|---|---|---|---|---|
| Wt% | 20.61 | 56.99 | 22.40 | 100 |

**Table S3** The content of each component of $MnO/CoMn_2O_4{\subset}GN$

| Element | Co K | Mn K | O K | C K | Total |
|---|---|---|---|---|---|
| Wt% | 9.33 | 20.4 | 14.29 | 55.98 | 100 |
